# Vaccination dropout rates among children aged 12-23 months in Democratic Republic of the Congo: a cross-sectional study

**DOI:** 10.1186/s13690-021-00782-2

**Published:** 2022-01-05

**Authors:** Harry-César Kayembe-Ntumba, Felly Vangola, Papy Ansobi, Germain Kapour, Eric Bokabo, Bien-Aimé Mandja, Didier Bompangue

**Affiliations:** 1 Ecology and Control of Infectious Diseases Unit, Department of Basic Sciences, Faculty of Medicine, University of Kinshasa, Kin XI, BP: 834 Kinshasa, Democratic Republic of the Congo; 2 Master of Ecology of Infectious Diseases, Natural Hazards and Risk Management, Faculty of Medicine, University of Kinshasa, Kinshasa, Democratic Republic of the Congo; 3 Chrono-Environnement Laboratory, CNRS, UMR 6249, University of Bourgogne Franche- Comté, Besançon, France

**Keywords:** Vaccination, Dropout rate, Predictors, Children, Mont Ngafula II health district, DRC

## Abstract

**Background:**

Overall, 1.8 million children fail to receive the 3-dose series for diphtheria, tetanus and pertussis each year in the Democratic Republic of the Congo (DRC). Currently, an emergency plan targeting 9 provinces including Kinshasa, the capital of the DRC, is launched to reinforce routine immunization. Mont Ngafula II was the only health district that experienced high vaccination dropout rates for nearly five consecutive years. This study aimed to identify factors predicting high immunization dropout rates among children aged 12-23 months in the Mont Ngafula II health district.

**Methods:**

A cross-sectional household survey was conducted among 418 children in June-July 2019 using a two-stage sampling design. Socio-demographic and perception data were collected through a structured interviewer-administered questionnaire. The distribution of 2017-2018 immunization coverage and dropout rate was extracted from the local health district authority and mapped. Logistic random effects regression models were used to identify predictors of high vaccination dropout rates.

**Results:**

Of the 14 health areas in the Mont Ngafula II health district, four reported high vaccine coverage, only one recorded low vaccine coverage, and three reported both low vaccine coverage and high dropout rate. In the final multivariate logistic random effects regression model, the predictors of immunization dropout among children aged 12-23 months were: living in rural areas, unavailability of seats, non-compliance with the order of arrival during vaccination in health facilities, and lack of a reminder system on days before the scheduled vaccination.

**Conclusions:**

Our results advocate for prioritizing targeted interventions and programs to strengthen interpersonal communication between immunization service providers and users during vaccination in health facilities and to implement an SMS reminder system on days before the scheduled vaccination.

## Background

Since the establishment of the Expanded Program on Immunization (EPI) in 1974, vaccines have had a significant impact on vaccine-preventable diseases (VPDs) by reducing morbidity, disability and mortality worldwide among children under five years of age [1, 2]. However, children remained not fully immunized with EPI-targeted disease vaccines in many parts of the world [3]. Although considered a key indicator of immunization program performance [4, 5], the third dose of vaccine against diphtheria, tetanus and pertussis (DTP3) has not been administered to 19.7 million children worldwide in their first year of life in 2019 [6]. The largest numbers of children who did not receive DTP3, 12.4 million (62%), were concentrated in ten countries: Nigeria, India, Pakistan, Indonesia, Ethiopia, the Democratic Republic of the Congo (DRC), Angola, Iraq, South Africa and Afghanistan [7]. Moreover, of all the children who did not complete the three-dose DTP series, 6.2 million (31%) started but did not complete the DTP series (dropout rate) [7].

In the DRC, 1.8 million children fail to receive the three-dose DTP series each year despite improvements in national DTP3 coverage from 25% to 1999 to 81% in 2018 through the partnership with the Vaccine Alliance [8]. These improvements have not made it possible to achieve the Congolese EPI goal of vaccinating >90% of children with three doses of DTP before their first birthday [9]. In addition, since 2004, the DRC has adopted the “Reaching Every District” approach, developed and introduced in 2002 by the World Health Organization (WHO), the United Nations Children’s Fund, and other partners to improve immunization systems in low coverage areas [10]. However, many children are still susceptible to VPDs and death. Poliomyelitis, yellow fever, and measles outbreaks have been reported across the country over the last ten years [11–15].

In 2018, the DRC launched an emergency plan to scale up routine immunization to vaccinate an additional 220.000 children within 18 months. This plan targeted 9 provinces: Ituri, Kasaï, Haut-Katanga, Mongala, Kwilu, Tanganyika, Tshuapa, Haut-Lomami, and Kinshasa (the capital of the DRC). Of the 35 health districts in Kinshasa, Mont Ngafula II was the only one with high vaccination dropout rates among children aged <12 months for nearly five consecutive years. Routine EPI reports showed dropout rates for the DTP3 dose of 15%, 19%, 10% and 11% in 2014, 2016, 2017 and 2018, respectively (Dataset V1). It is therefore necessary to develop appropriate strategies to improve routine immunization activities.

The present study aimed to identify predictors of high vaccination dropout rates among children aged 12-23 months in the Mont Ngafula II health district.

## Methods

### Study area

**Fig. 1:**
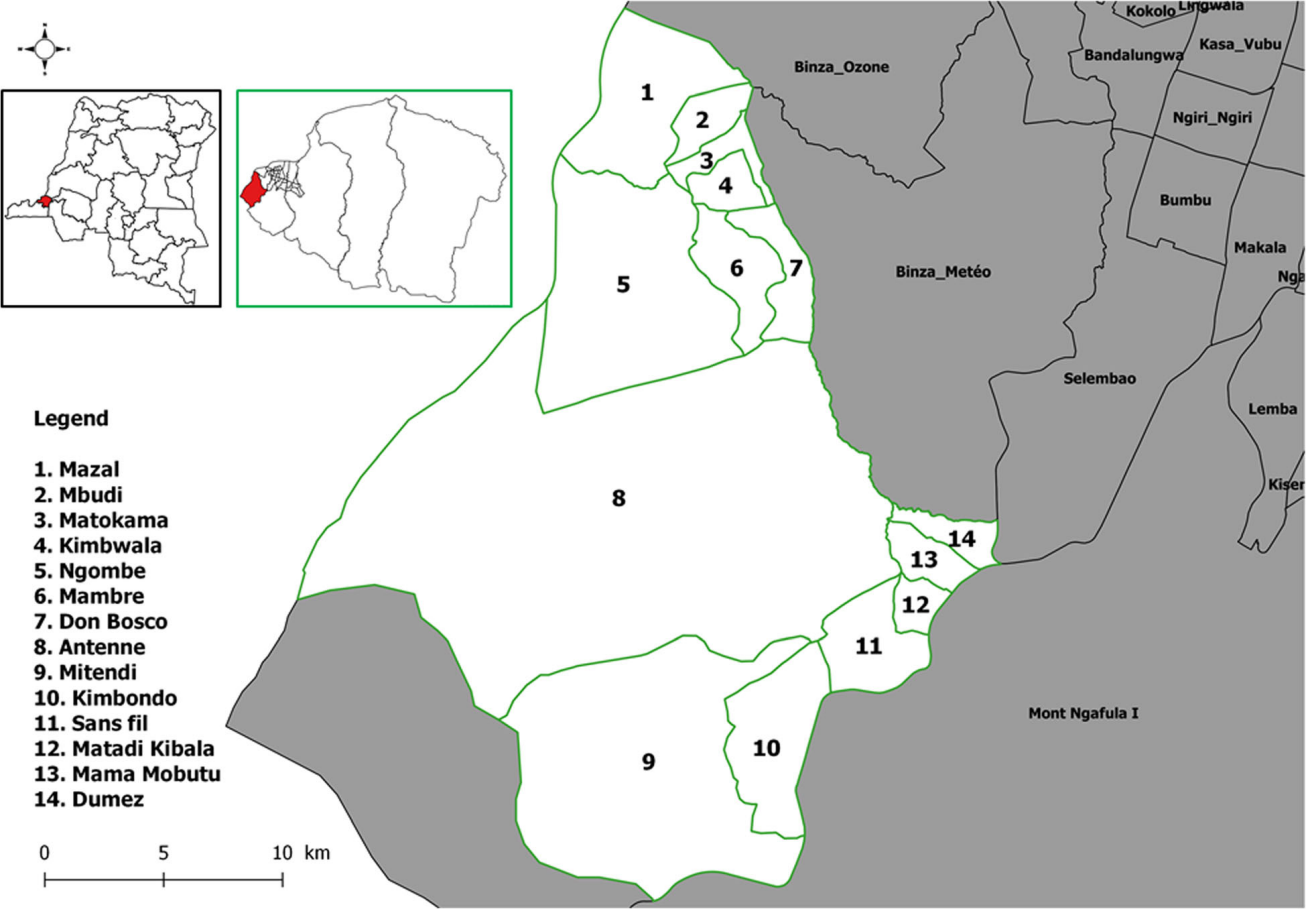
Study area.

The Mont Ngafula II health district is located in the western part of Kinshasa. It shares borders with five districts: Binza Ozone, Binza Météo, Selembao, Mont Ngafula I, and Masa (in Kongo central province). This health district is subdivided into 14 health areas: Antenne, Don Bosco, Kimbwala, Kimbondo, Maman Mubutu, Matadi Kibala, Mambre, Matokame, Mazal, Mitendi, Ngombe, Mbudi and Sans Fil (Fig. 1). It has a general pediatric hospital, 107 health centers including 22 health posts. Only 14 health centers deliver EPI vaccines to the target population groups. The main local economic activities are based on agriculture, livestock, and fishery products.

### Study design and sampling procedure

A cross-sectional household survey was conducted. All mothers/caregivers with children aged 12–23 months, between the 1st of June and the 1st of July 2019, were eligible. Since the exhaustive number of households and children aged 12–23 months was not available, we referred to the WHO vaccination coverage cluster surveys to perform a two-stage sampling design [16]. As recommended to select an appropriate sample size by ensuring a maximum absolute confidence interval width of ± 10% at fairly low vaccination coverage, 30 clusters were first sampled using probability proportional to their size, based on estimated population data per village/neighborhood provided by the health district authority. The sample size was determined using the following formula: *N*= Z^2^×p(1-p)/d^2^, where N is the sample size, Z (1.96) is the 2-sided level of significant, p is the immunization coverage of the country (45%) as proposed in the study of Baguune at al. [17], and d is the desired width of the confidence interval. Considering a non-response rate of 10%, we found a total sample size of 418 children aged 12–23 months. At the second stage, we used the EPI random walk method [18] to select 14 children from each of the 30 clusters. The starting point was considered the first household randomly selected at the geographic center of each cluster using the spinning bottle method, and then we continued to the nearest household in the same direction until 14 eligible children were obtained.

### Data collection

A structured questionnaire translated into the local language was used to collect data on sociodemographic characteristics (children, respondents and household) and respondents’ perceptions. Interviewers were previously recruited and trained on the study protocol and the questionnaire use. After the training, a pre-test was conducted in the field to check the reliability and validity of our study tools. The questionnaire included information on socio-demographic characteristics, child immunization status, and perception of respondents (mothers/caregivers) on the organization of vaccination in health facilities. Door-to-door visits were conducted and only consenting respondents of selected children participated in the survey. In case where two or more children aged 12–23 months were found in the same household, the youngest child was selected [18]. Information on children’s immunization, such as dates and doses of each vaccine received, was obtained from vaccination cards or respondents’ recall if the card was not available. The data collected was then recorded on Microsoft Excel® 2013 files.

Annual routine immunization coverage and dropout rate data were extracted from the 2017-2018 database collected at the health area scale provided by the local health authority. They were also recorded in Microsoft Excel® 2013 (Dataset V2). According to the Congolese routine immunization schedule (Table 1), the dropout rate was considered the percentage of children aged 12-23 months who received the 1st but not the 3rd dose of DTP: [(first dose – last dose)/first dose] ×100 [4].

**Table 1:** Routine immunization schedule in the DRC

Timing	Vaccine
Birth	Bacillus Calmette-Guerin (BCG) and Oral Polio Vaccine (OPV)
6, 10, 14 weeks	OPV, Pentavalent vaccine (DTP, Haemophilus Influenzae type B and Hepatitis B), Pneumococcal Vaccine, and Rotavirus vaccine each time
9 months	Measles and Yellow fever vaccines

### Data analysis

We used data obtained from the Mont Ngafula II health authority to map the annual geographic distribution of routine immunization coverage and dropout rates at the health area scale using QGIS 2.14.4 (Open-Source Geospatial Foundation). The Mont Ngafula II health district shapefile was provided by the Directorate of Disease Control of the DRC Ministry of Public Health (validated by WHO DRC) and used to generate the map. The latter allowed to assess the dynamic system of vaccination services at the health area level.

Data collected through the cross-sectional household survey were used to identify factors predicting high vaccination dropout rates among children aged 12-23 months in the Mont Ngafula II health district. Descriptive statistics such as frequencies and percentages were calculated and presented into tables. Differences in children, respondents, household characteristics, and respondents’ perceptions were tested by the Chi-square test. Since our data were clustered at the health area scale, we performed logistic random effects regression models using the R package glmmML to measure the association between independent predictors and immunization dropout. The dependent variable “dropout” was coded as 0 for children who received the three-dose DTP series and 1 for children who received the DTP1 but not the DTP3. The explanatory variables were also summarized into two categories: sex of child (0 = male; 1 = female), birth order (0 = ≤ 2nd; 1 = ≥ 3rd), card possession (0 = yes; 1 = no), age of respondent (0 = ≤ 19 years; 1 = > 19 years), education level (0 = ≥ secondary; 1 = ≤ primary), occupation (0 = no; 1 = yes), place of residence (0 = urban; 1 = rural), type of family (0 = 2-parents; 1 = single parent), number of children (0 = ≤ 2 children; 1 = ≥ 3 children), attitude of health staff (0 = positive; 1 = negative), availability of seats (0 = yes; 1 = no), respect of the order of arrival (0 = yes; 1 = no), waiting time (0 = < 1 h; 1 = ≥1 h), reminder system (0 = yes; 1 = no). The final multivariate logistic random effects model included variables that were selected by a subsequent backward selection based on the results of the univariate analysis and then the multivariate model for each section of the household survey. At each step, variables with *p* ≥ 0.20 were excluded until only variables with a *p* < 0.20 remained. All statistical analyses were performed with R® version 3.6.1.

## Results

A total of 418 children and their mother/caregivers were assessed in the study. Overall, 9 out of 10 children possessed vaccination card, 50.2% were female, and 53.6% were the 3rd or later born in the family. The most represented age group of the respondents was 20–29 years (50%), followed by ≥ 30 years (42.3%). All respondents had formal education, 69.9% at secondary level and 12.4% at tertiary level. More than half (54.5%) of the respondents were unemployed. Nearly, two-third (63.6%) of the respondents lived in urban areas and 92.1% had less than 3 children in their households (Table 2).

**Table 2:** Sociodemographic characteristics of participants, Mont Ngafula II health district, DRC, 2019

Variables		Number	Percent (%)
*Children characteristics*			
Sex	Female	210	50.2
	Male	208	49.8
Birth order	≤2nd	194	46.4
	≥3rd	224	53.6
Card possession	No	42	10.0
	Yes	376	90.0
*Respondents’ characteristics*			
Age group	≤ 19 years	32	7.7
	20-29 years	209	50.0
	≥ 30 years	177	42.3
Occupation	Professional	28	6.7
	Trader	104	24.9
	Unemployed	228	54.5
	Other	58	13.9
Education level	Primary	74	17.7
	Secondary	292	69.9
	Tertiary	52	12.4
*Household characteristics*			
Place of residence	Rural	152	36.4
	Urbain	266	63.6
Type of family	Single parent family	78	18.7
	2-parents family	340	81.3
Number of children	≤2	385	92.1
	≥3	33	7.9

Table 3 shows the respondents’ perceptions on the organization of vaccination in health facilities of the Mont Ngafula II health district. The majority indicated a good attitude of health staff during vaccination (94.0%), the availability of seating (95.0%), the respect of the order of arrival during vaccination (90.0%), and a long waiting time (83.0%) during vaccination in health facilities. More than half (58.9%) of the participants reported the absence of a reminder system on days before the scheduled vaccination.

**Table 3:** Respondents’ perceptions on the organization of vaccination in health facilities, Mont Ngafula II health district, DRC, 2019

Perceptions	Number	Percent (%)
Attitude of health staff during vaccination in health facilities		
Bad	25	6.0
Good	393	94.0
Availability of seats during vaccination		
No	21	5.0
Yes	397	95.0
Respect of the order of arrival during vaccination		
No	42	10.0
Yes	376	90.0
Waiting time during vaccination in health facilities		
< 1 h	71	17.0
≥ 1 h	347	83.0
Existence of a reminder system on days before the scheduled vaccination		
No	246	58.9
Yes	172	41.1

### Spatial distribution of routine immunization coverage and dropout rate

**Fig. 2:**
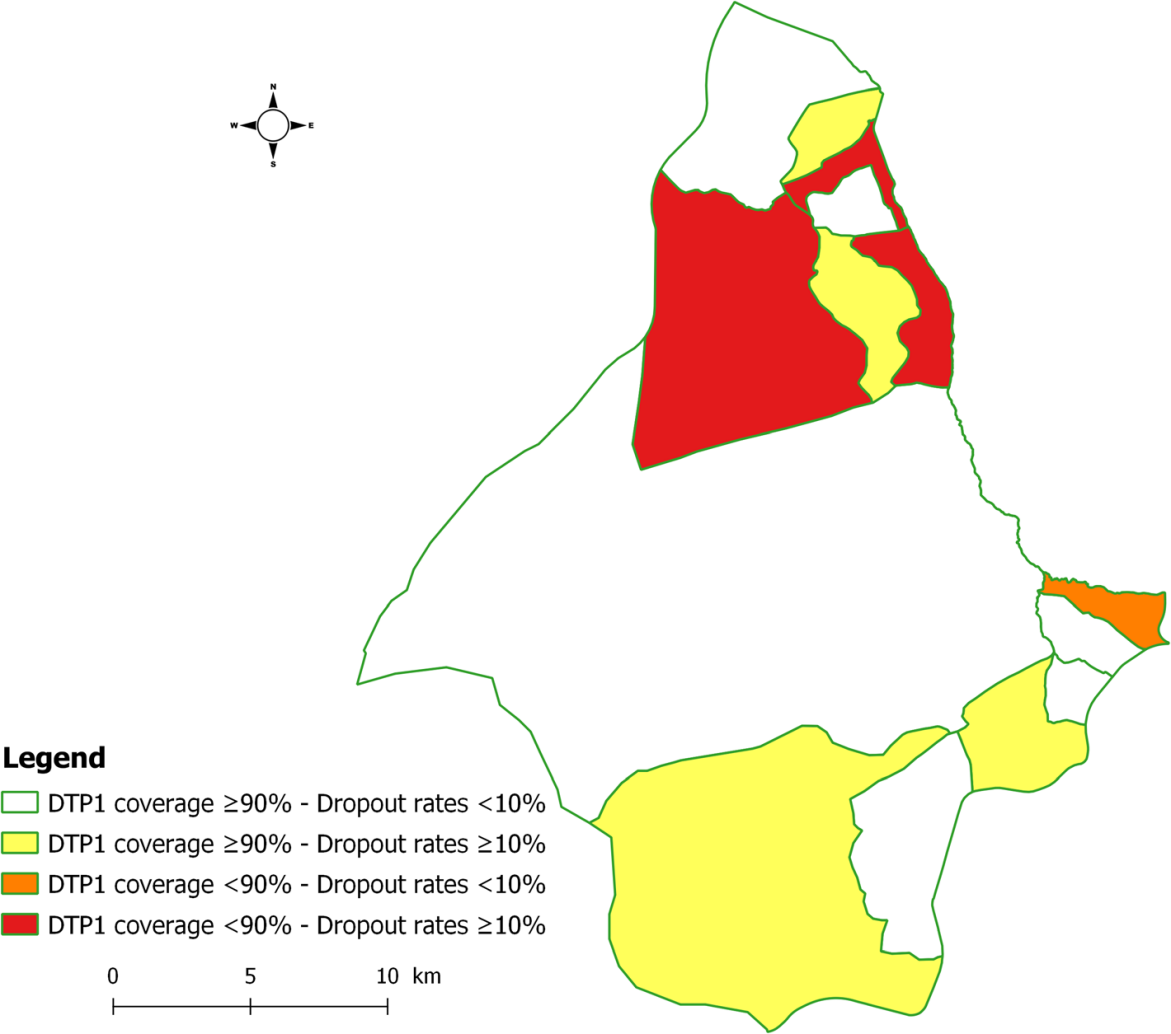
Spatial distribution of 2017-2018 routine immunization coverage and dropout rate.

Figure 2 summarizes the spatial distribution of 2017-2018 routine immunization coverage and dropout rate. Four health areas reported high vaccine coverage and high dropout rate: Mitendi, Sans fil, Mbudi and Mambre. Three health areas recorded low vaccine coverage and high dropout rate: Matokama, Ngombe and Don Bosco. Only one health area reported low vaccine coverage and low dropout rate: Dumez.

### Sociodemographic characteristics and perceptions of participants associated with dropout status

Table 4 compares sociodemographic characteristics and perceptions of participants by vaccination dropout status (based on vaccination cards and respondents’ recall). Children with vaccination cards had a lower dropout rate compared to those without (42.8% vs. 64.3%). There were no statistical differences in other sociodemographic characteristics. Children of respondents who reported positive attitude of health staff, availability of seating places and respect of the order of arrival during vaccination in health facilities were more likely fully immunized. The absence of a reminder system on days before the scheduled vaccination was found associated with higher dropout rate (Table 4).

**Table 4:** Comparison of sociodemographic characteristics and perceptions based on vaccination dropout status using Chi-square test, Mont Ngafula II health district, DRC, 2019

Variables		No Dropout		Dropout		Total	*p*
		**N**	**%**	**N**	**%**	**N**	
*Children characteristics*							
Child sex	Female	115	54.8	95	45.2	210	0.914
	Male	115	55.3	93	44.7	208	
Birth order	≤2nd	101	52.1	93	47.9	194	0.257
	≥3rd	129	57.6	95	42.4	224	
Card possession	No	15	35.7	27	64.3	42	0.008
	Yes	215	57.2	161	42.8	376	
*Respondents’ characteristics*							
Age	≤ 19 years	16	50.0	16	50.0	32	0.148
	20-29 years	124	59.3	85	40.7	209	
	≥ 30 years	90	50.8	87	49.2	177	
Education level	Primary	38	51.4	36	48.6	74	0.247
	Secondary	168	57.5	124	42.5	292	
	Tertiary	24	46.2	28	53.8	52	
Occupation	Professional	16	57.1	12	42.9	28	0.718
	Trader	62	59.6	42	40.4	104	
	Unemployed	121	53.1	107	46.9	228	
	Other	31	53.4	27	46.6	58	
*Household characteristics*							
Place of residence	Rural	75	49.3	77	50.7	152	0.078
	Urbain	155	58.3	111	41.7	266	
Type of family	Single parent family	42	53.8	36	46.2	78	0.817
	2-parents family	188	55.3	152	44.7	340	
Number of children	≤2	214	55.6	171	44.4	385	0.431
	≥3	16	48.5	17	51.5	33	
*Respondents’ perceptions*							
Attitude of health staff	Negative	7	28.0	18	72.0	25	0.005
	Positive	223	56.7	170	43.3	393	
Availability of seats	No	2	9.5	19	90.5	21	<0.001
	Yes	228	57.4	169	42.6	397	
Respect of the order of arrival	No	8	19.0	34	81.0	42	<0.001
	Yes	222	59.0	154	41.0	376	
Waiting time	>1 h	188	54.2	159	45.8	347	0.443
	<1 h	42	59.2	29	40.8	71	
Reminder system	No	116	47.2	130	52.8	246	<0.001
	Yes	114	66.3	58	33.7	172	

### Predictors of high vaccination dropout rates

Table 5 shows independent predictors of immunization dropout identified at the final multivariate logistic random effects regression model. Residing in rural areas was associated with high vaccination dropout (aOR = 1.87; 95% CI = 1.12-3.14). Unavailability of seats (aOR = 7.30; 95% CI = 1.30-40.87) and non-compliance with the order of arrival (aOR = 3.42; 95% CI = 1.36-8.61) during vaccination in health facilities, and lack of a reminder system on days before the scheduled vaccination (aOR = 2.04; 95% CI = 1.28-3.24) were perceived significantly associated with high dropout rate.

**Table 5:** Predictors of high immunization dropout rate identified by multivariate logistic random effects regressions models

Variables	Model 1 aOR (95% CI)	Model 2 aOR (95% CI)	Model 3 aOR (95% CI)	Model 4 aOR (95% CI)
**Sociodemographic characteristics**				
*Children characteristics*				
Card possession (No vs. Yes)	2.28 (1.13- 4.60)			2.04 (0.93- 4.49)
*Household characteristics*				
Place of residence (Rural vs. Urban)		1.92 (1.18-3.12)		1.87 (1.12-3.14)
**Respondents’ perceptions**				
Availability of seats (No vs. Yes)			7.91 (1.49-41.90)	7.30 (1.30-40.87)
Respect of the order of arrival (No vs. Yes)			3.52 (1.42-8.73)	3.42 (1.36-8.61)
Reminder system (No vs. Yes)			2.12 (1.34-3.34)	2.04 (1.28-3.24)
Random effects variance (SE)	0.52*(0.16)	0.63*(0.18)	0.56*(0.18)	0.63*(0.19)
Model fit AIC	564.6	562.9	531	526.7

aOR, adjusted odds ratio; SE, Standard Error; AIC, Akaike Information Criterion

Model 1 multivariate regression for children characteristics.

Model 2 multivariate regression for household characteristics.

Model 3 multivariate regression for respondents’ perceptions.

Model 4 final multivariate logistic random effects regression (chidren and household characteristics, and respondents’ perceptions).

## Discussion

This study showed that half of the health areas in the Mont Ngafula II health district had high dropout rates. More than two-fifths also recorded low vaccine coverage, while only one health area reported exclusively low vaccine coverage. Living in rural areas, on the one hand, unavailability of seats and non-compliance with the order of arrival during vaccination in health facilities, and lack of a reminder system on days before the scheduled vaccination, on the other hand, were significantly associated with high dropout rates among children aged 12-23 months.

Concerning the local system of vaccination services, the poor utilization of vaccine services emerged as the main health concern, followed by the poor access at the health area level. More than half of the health areas reported good access but poor utilization of vaccination services (high vaccine coverage and high dropout rate), while two-fifths reported both poor access and poor utilization vaccination services (low vaccine coverage and high dropout rate). Furthermore, only one health area reported both poor access and high utilization of vaccination services (low vaccine coverage and low dropout rate). Thus, these results may represent a useful indicator for assessing the risks of immunization services in the study area.

In the present study, children from rural areas were more likely to drop out than those from urban areas. However, discordant results have been reported in the literature. Living in rural areas was associated with breaks in childhood vaccination in India [19], whereas this characteristic was found to be protective in a multilevel analysis involving 24 African countries [20]. These observed discrepancies call into question the urban advantage, probably due to the growth of the urban population and the resulting difficulties in accessing health services [19, 21–23]. Children without immunization card have higher susceptibility to drop out from vaccination services [17, 24–26]. This may be related to the perception of the health staff’s attitudes. It has been suggested that the negative attitudes of some health workers, who are unable to provide new vaccination cards if lost, could induce mothers’ reluctance to have their children revaccinated at health facilities [17]. We therefore believe that the positive attitudes of health personnel can enhance interpersonal communication and trigger subsequent use of immunization services.

This study also highlighted that unavailability of seats and non-compliance with the order of arrival during vaccination in health facilities, as well as the lack of a reminder system on days before the scheduled vaccination, were perceived to be associated with high immunization dropout rates. These results are consistent with findings from other studies conducted in Zimbabwe [27] and Kenya [28, 29], in which complete immunization was significantly higher among children of mothers/caregivers who received short message services (SMS) reminders than those in control groups. Thus, our findings support the hypothesis that implementing and expanding a reminder system using SMS-type messages may contribute to significant improvements in immunization services in our context.

Other sociodemographic characteristics considered in the present study were not statistically associated with dropout rates, whereas they were found as predictors of incomplete vaccination elsewhere. Children (birth order and sex), parents (education level and religion), and household (number of children) characteristics were significantly related to children’s immunization status in several African studies [17, 24, 28, 30–36]. Growing number of children, sex discrimination, low parents’ education level, and misconceptions from certain religious groups may affect vaccination uptake and lead to immunization dropout [17, 25, 27]. The contrasting results observed in our study represent the first limit. This may be due to our sample size and sampling procedure, which were smaller and somewhat different from other studies, respectively [16, 23, 25].

Another study limitation is that the determinants of vaccination dropout rates were assessed from the perspective of the users of vaccination services. The perspective of health systems and providers was not included in our study. However, the determinants of childhood immunization coverage are complex and interrelated. Vaccination depends on a real need for vaccines and a seek for health service from users, and on an offer of quality vaccine services from providers under optimal technical, logistical, and operational conditions [37].

Nonetheless, there has been a limited number of research in DRC on vaccination dropout [38]. To our knowledge, the present study is the first conducted at both the fine spatial and individual levels to understand the main challenges of immunization services and to identify factors related to vaccination dropout among children aged 12-23 months. An additional strength of this study is that our results can be extrapolated to the DRC population because the survey used a probability sampling technique and the minimum sample size was calculated by taking into account the formula required to allow each person to be included in the sample.

## Conclusions

The poor utilization of immunization services emerged as the main health concern, followed by the poor access in the Mont Ngafula II health district. Living in rural areas, unavailability of seats and non-compliance with the order of arrival during vaccination in health facilities, and the absence of a reminder system on days before the scheduled vaccination were the predictors of high vaccination dropout rates among children aged 12-23 months. These results highlight the need to prioritize targeted vaccination interventions and programs that will strengthen interpersonal communication between immunization providers and users during vaccination in health facilities. In addition, our findings advocate for the implementation and extension of a reminder system using SMS reminders on days before the scheduled vaccination. Adopting such an approach would be even more beneficial for rapid immunization in the fight against child morbidity and mortality, especially since the cost has proven to be affordable [27, 39, 40].

Further studies on determinants attributable to health workers and immunization services will help to explore in depth the factors associated with routine vaccination dropout rates among children aged 12-23 months.

## Data Availability

The datasets generated and/or analysed during this study are available in the: [Vaccination_dropout_rates_Mont_Ngafula_II_Dataset] repository, [https://www.editorialmanager.com/aoph/download.aspx?id=33405&guid=289f34cc-a2e1-45de-b862-f013c6f0bcf9&scheme=1] [Dataset V1] repository, [https://www.editorialmanager.com/aoph/download.aspx?id=33406&guid=453a4c34-238a-4d2c-ab4a-7548220d5a91&scheme=1] [Dataset V2] repository, [https://www.editorialmanager.com/aoph/download.aspx?id=33407&guid=8a0acba5-461b-4eb0-ad95-86eac6486ba2&scheme=1]

## Abbreviations

95% CI: 95% confidence interval
aOR: Adjusted Odds Ratio
DRC: Democratic Republic of the Congo
DTP: Diphtheria, tetanus and pertussis vaccine
EPI: Expanded Program on Immunization
OPV: Oral Polio Vaccine
SMS: Short message services
VPDs: Vaccine-preventable diseases
WHO: World Health Organization
